# The Use of Population Pharmacokinetics to Extrapolate Food Effects from Human Adults and Beagle Dogs to the Pediatric Population Illustrated with Paracetamol as a Test Case

**DOI:** 10.3390/ph17010053

**Published:** 2023-12-28

**Authors:** Elke Gasthuys, Louis Sandra, Marina Statelova, Maria Vertzoni, An Vermeulen

**Affiliations:** 1 Department of Bioanalysis, Faculty of Pharmaceutical Sciences, Ghent University, Ottergemsesteenweg 460, 9000 Ghent, Belgium; louis_sandra@hotmail.com (L.S.); anmc.vermeulen@ugent.be (A.V.); 2 Department of Pharmacy, National and Kapodistrian University of Athens, Panepistimiopolis, Zografou, 157 84 Athens, Greece; marina.statelova@novartis.com (M.S.); vertzoni@pharm.uoa.gr (M.V.); 3 Analytical Research and Development, Global Drug Development, Novartis Pharma AG, Fabrikstrasse 2, 4056 Basel, Switzerland; 4 Clinical Pharmacology and Pharmacometrics, Janssen R&D, A Division of Janssen Pharmaceutica NV, Turnhoutseweg 30, 2340 Beerse, Belgium

**Keywords:** paracetamol, pediatrics, beagle dogs, adults, food effect, population pharmacokinetics

## Abstract

To date, food–drug interactions in the pediatric population remain understudied. The current food effect studies are mostly performed in adults and do not mimic the real-life situation in the pediatric population. Since the potential benefits of food effect studies performed in pediatrics should be counterbalanced with the burden that these studies pose to the patients, alternative research strategies should be evaluated. The present study aimed to evaluate whether population pharmacokinetics (popPK) using data in beagle dogs and human adults could reliably assess food effects relevant for the pediatric population. PopPK was utilized to understand the performance of paracetamol under different dosing conditions (when the participants were fasted, with a reference meal, and with infant formula) in human adults (*n* = 8) and beagle dogs (*n* = 6) by constructing models to derive the pharmacokinetic parameters and to evaluate the food effects in both species. A two-compartment model with a single input function for the absorption phase best described the profiles of paracetamol in the beagle dogs. In the human adults, a one-compartment model with a dual input function for the absorption phase best described the data. The simulated profiles for the different dosing conditions demonstrated that both the human adults’ and beagle dogs’ simulations were able to acceptably describe the plasma concentration–time profiles of paracetamol observed in a representative pediatric population, which opens up perspectives on pediatric-relevant food effect predictions. However, the obtained results should be carefully interpreted, since an accurate validation of these findings was not possible due to the scarcity of the literature on observed pediatric data.

## 1. Introduction

The oral dosing of drugs is the preferred route of administration in the pediatric population. When administering drugs orally to this population, parents and caregivers often face certain challenges, such as the availability of the correct dosing regimens, the availability of pediatric-adapted formulations, the lack of information about palatability, etc. [1]. Children are often not able or willing to take a drug due to its swallowability or taste, resulting in parents and caregivers using food to improve drug acceptance and therapeutic adherence [2]. Moreover, pediatric patients might receive foods/snacks more frequently than adults, resulting in not “truly” fasted conditions. Therefore, pediatric patients are often in a non-fasting/fed state when drugs are administered orally. However, to date, food–drug interactions in the pediatric population remain largely understudied [3]. To stimulate food–drug interaction studies, regulatory authorities, such as the European Medicines Agency (EMA) and the Food and Drug Administration (FDA), have developed guidelines on how to design and perform food effect studies [4,5]. However, these guidelines mostly apply to the adult situation, where a high-fat, high-caloric solid–liquid meal (800 to 1000 calories) is administered half an hour before the drug product of interest, in order to achieve the maximum impact on the gastrointestinal physiology. This situation does not mimic the real-life situation in the pediatric population, especially in the young pediatric age groups, raising the question of whether the study protocol should be adapted to more reliably predict food–drug interactions in this population [3]. Ideally, food-effect studies should be performed in the intended population; however, the potential benefits of such studies should be counterbalanced with the burden that these studies pose to the pediatric patients. Moreover, the practical and ethical challenges accompanying these studies render it difficult to perform such studies in pediatrics. Therefore, alternative research strategies deserve careful evaluation. One alternative study design to evaluate food effects in the pediatric populations is to adapt the standard high-fat, high-caloric meal to a pediatrically relevant meal (by adapting the meal quantity or content) and to extrapolate the adult data to the pediatric population using modelling and simulation tools (population pharmacokinetics (popPK) and/or physiologically based pharmacokinetics (PBPK)). The latter research strategy was also suggested by a recent FDA guideline, which stated that the composition of the food (the type and meal quantity) should be adapted for the target pediatric population [4]. 

Paracetamol (acetaminophen) is a first-line antipyretic and analgesic that is commonly used in the pediatric population [6]. The mechanism of action of paracetamol still needs to be fully established, but it comprises, inter alia, cyclooxygenase inhibition as well as involvement in the endocannabinoid system and the serotonergic pathway [7]. Initially, paracetamol was classified as a BCS class III compound (Biopharmaceutical Classification System) with high solubility and low permeability, but this was later adapted to a BCS class I, since it demonstrates high solubility and complete absorption (>85%) [8,9]. Paracetamol is commonly administered irrespective of the prandial state. It has been demonstrated that concomitant food intake does not significantly affect the overall oral bioavailability, but it can lead to a delayed paracetamol absorption (a prolonged Tmax (time to maximum plasma concentration) and a reduced Cmax (maximum plasma concentration) [10,11,12]. Moreover, paracetamol has been utilized as a marker of gastric emptying [13].

The aim of this present study was to determine whether a population pharmacokinetic (popPK) approach could be suitable to reliably assess the food effects that are relevant for the pediatric population using human adult and beagle dog data as inputs. PopPK models describing the PK of paracetamol in both species were constructed to gain more insights into the drug’s performance based on different dosing conditions (fasted, reference meal (RM), and infant formula (IF)) and to verify these insights using the available (published) data on the pediatric population. The results obtained with the present popPK analysis were compared with a similar PBPK exercise performed by Statelova et al. [14,15].

## 2. Results

### 2.1. Study Data, Model Development and Evaluation

**Figure 1:**
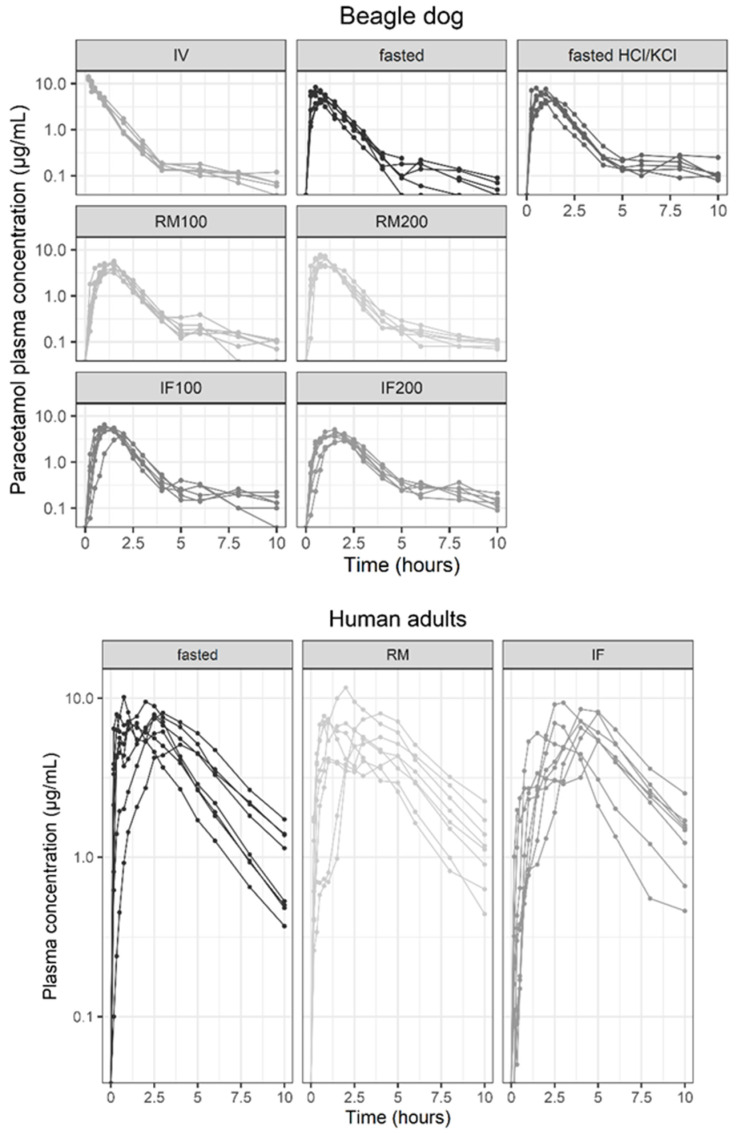
Paracetamol plasma concentration–time curves in beagle dogs [14] and human adults [16]. RM: reference meal; IF: infant formula; RM200: 100 g (200 kcal); RM100: 50 g (100 kcal); IF100: 150 mL (100 kcal); INF200: 300 mL (200 kcal); RM: 990 kcal; and IF: 520 kcal.

The present datasets included 575 (six beagle dogs, eight occasions and 360 (eight human adults, three occasions) plasma concentration datapoints. The plasma concentration–time curves of the paracetamol in the beagle dogs and the human adults for the different dosing conditions are depicted in Figure 1.

When visually inspecting the plasma concentration–time curves of the orally administered paracetamol in the human adults, a rather atypical absorption profile could be observed. In some of these profiles, a double absorption peak or shoulder was noted, which was more apparent for the RM (four out of the eight subjects) in comparison to the other dosing conditions (fasted: two out of the eight subjects; IF: one out of the eight subjects). The double peaking could not be attributed to only one specific subject. One subject showed a double peak in the plasma concentration–time curve of the fasted and RM, and one other subject showed a double peak in the plasma concentration–time curve of the RM and IF. The other subjects who demonstrated a double absorption peak only had one double absorption peak present in either dosing condition. This double peak phenomenon was not observed in the plasma concentration–time profiles of the beagle dogs.

In the beagle dogs, significant differences between the different dosing conditions could be observed (Table 1), whereas in the human adults these differences were less apparent (Table 2). A delayed absorption with a prolonged T_max_ and a reduced C_max_ was noted when the beagle dogs received the RM100 (50 g, 100 kcal), the IF100 (150 mL, 100 kcal), and the IF200 (300 mL, 200 kcal), although the total exposure (AUC_0-inf_) was not significantly different in comparison to the fasted condition (with and without pre-treatment with HCl/KCl). This was in contrast to the RM200 (100 g, 200 kcal), where the total exposure was significantly higher (*p* < 0.05) than when the beagle dogs were fasted, but the absorption did not appear delayed. When comparing the low amount/low caloric with the high amount/high caloric RM, a significant difference (*p* < 0.05) in the C_max_ (RM100 < RM200), T_max_ (RM100 > RM200), and AUC_0-inf_ (RM100 < RM200) could be observed. The significant differences observed between the RM100 and the RM200 were expected and are supported by a study performed by Lentz et al. [17]. When looking at the IF, only the C_max_ differed significantly (IF100 > IF200) (*p* < 0.05). When comparing the same amount/caloric content of the RM and the IF, no significant differences between the RM100 and the IF100 could be detected. By contrast, a prolonged T_max_ and a reduced C_max_ could be observed when the beagle dogs received the IF200 compared to when they received the RM200. When evaluating the plasma concentration–time curves in the human adults, only a significantly lower T_max_ (*p* < 0.05) could be observed when the human adults were fasted instead of receiving an IF.

**Figure 2:**
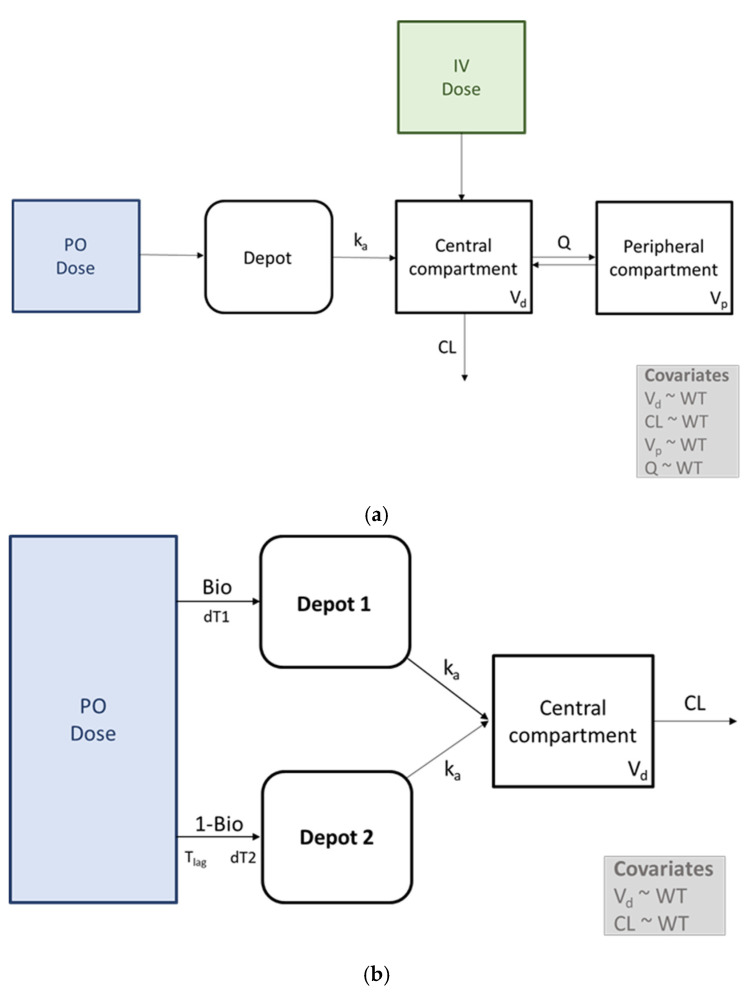
Graphical representation of the final model structure to describe the pharmacokinetics of paracetamol in the beagle dogs (**a**) and the human adults (**b**). k_0_ = zero-order input rate; k_a_ = first-order absorption rate constant; V_d_ = volume of distribution of the central compartment; Q = intercompartmental flow; V_p_ = volume of distribution of the peripheral compartment; CL = total body clearance; Bio = fraction of the absorbed dose; dT1 = duration of zero-order input into the dosing compartment 1; dT2 = duration of zero-order input into the dosing compartment 2; T_lag_ = lag time, PO = per os; and IV = intravenous.

A two-compartment model with a single input function for the absorption phase best described the plasma concentration–time profiles of the paracetamol in the beagle dogs (Figure 2a). The absorption was described using a sequential zero-order absorption (k_0_) into the depot compartment followed by a first-order absorption (k_a_) into the central compartment. The final model was parameterized using the absolute oral bioavailability (F), since the data were collected after intravenous (IV) dosing in the beagle dogs group. The final model was further improved by accounting for a significant covariate effect of the WT on the CL, V_d_, V_p_, and Q. In the human adults group, a one-compartment model with a dual input function for the absorption phase best described the paracetamol data (Figure 2b). A dual input function was applied where a fraction of the dose was absorbed (zero-order absorption) into a first (Bio, dT1) and second (1-Bio, dT2) dosing compartment, respectively. For the second dosing compartment (1-Bio), a lag time was implemented. Following the zero-order release into the respective dosing compartments, the first-order absorption (k_a_) into the central compartment happened. Apparent parameters relative to F were obtained for the CL and V_d_, since in the human adults no IV administration of paracetamol was performed within the same study. Accounting for a significant covariate effect of the WT on the CL and V_d_ further improved the model fit. The final PK model parameter estimates, the between-subjects/inter occasion variability in these parameters, and the log-likelihood estimation for both species are summarized in Table 3. 

**Figure 3:**
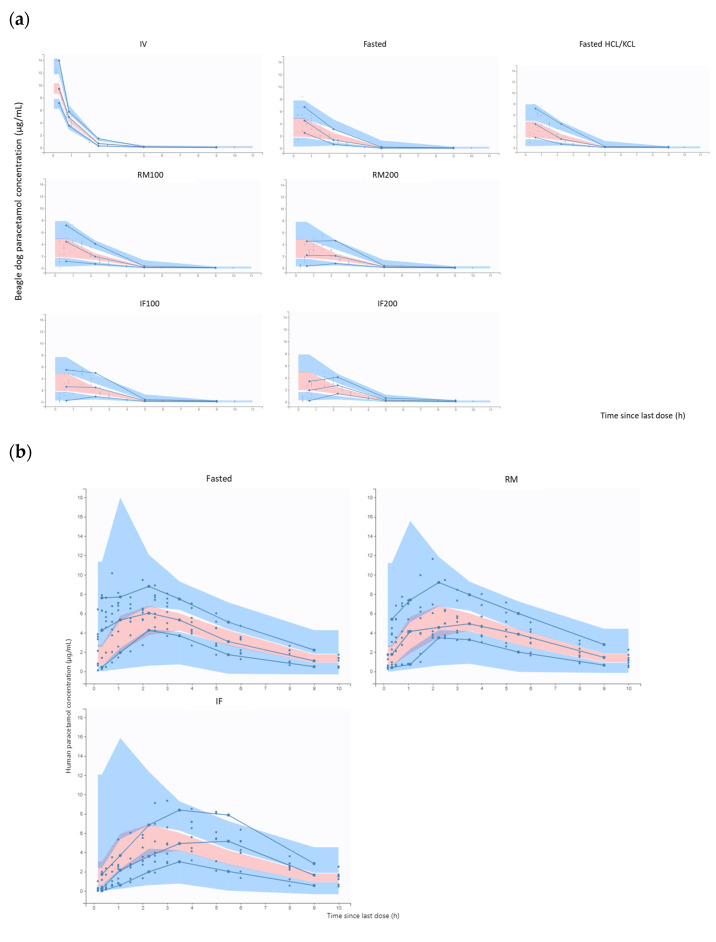
Prediction-corrected visual predictive check (pcVPC) plots in the beagle dogs (**a**) and the human adults (**b**) for the different occasions. Blue circles represent the observed data (18); blue full lines are the 10th, 50th and 90th percentiles; shaded areas are the 90% confidence intervals of the corresponding percentiles. RM: reference meal; IF: infant formula; RM200: 100 g (200 kcal); RM100: 50 g (100 kcal); IF100: 150 mL (100 kcal); INF200: 300 mL (200 kcal); RM: 990 kcal; and IF: 520 kcal.

In general, the data were adequately described with the final obtained models (Figure S1, supplementary data). A visual inspection of the goodness-of-fit plots for the final population PK models showed no apparent bias for the predictions (Figures S2 and S3, supplementary data). Since most of the observed plasma concentrations were within the 90% prediction interval of the pcVPC for the different dosing conditions (Figure 3), the predictive performance of the model was confirmed.

### 2.2. Simulations

**Figure 4:**
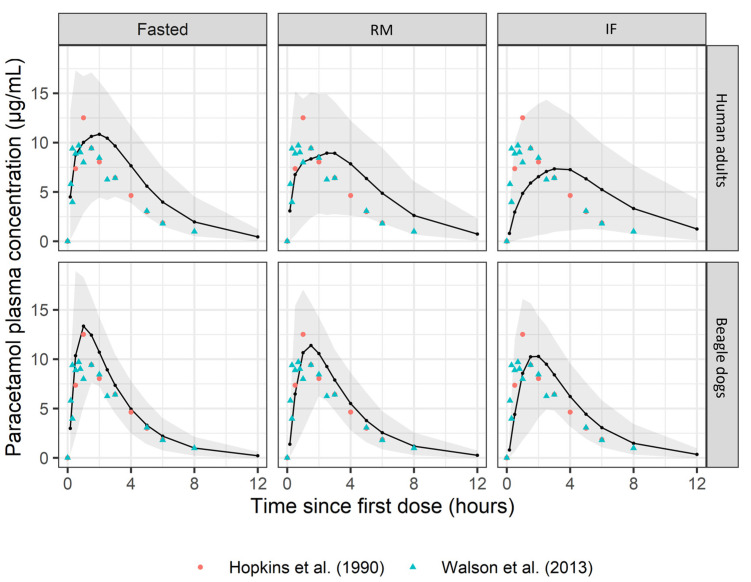
Simulations of plasma concentration–time profiles for the paracetamol using the human adult parameters (upper panel) and the beagle dog PK parameters (lower panel) dosed in different dosing conditions (fasted, reference meal, and infant formula). The shaded areas represent the 90% prediction interval. Symbols show the observed data from the respective clinical studies from the literature. The prandial state in the study of Hopkins et al. [18] and Walson et al. [19] was not reported (dose: 15 mg/kg).

A visual inspection of the simulated profiles for the different dosing conditions (Figure 4) demonstrated that both the simulation based on the human adult PK parameters (upper panel) and the simulation using the absorption parameters of the beagle dogs, combined with the allometrically scaled disposition parameters (lower panel), were able to acceptably describe the plasma concentration–time profiles of the paracetamol in a representative pediatric population. It was decided not to stratify the beagle dogs by caloric content, since no significant covariate related to food could be retained in the final popPK model.

Upon initial investigation, the predictions based on the beagle dog PK input parameters were slightly better than those based on the human adult PK parameters. As can be derived from Figure 4, the C_max_ was lower and the T_max_ occurred later when looking at the simulated human adult plasma concentration–time curves in comparison to the observations. The latter was especially observed in the RM and IF dosing conditions. However, it should be noted that only one observation fell outside the 90% prediction interval, whereby it was concluded that the human adult data reasonably predicted the pediatric paracetamol plasma concentration–time curves. The discrepancy between the observed and the predicted values could be attributed to the variability in the data as well as the lack of information about the prandial status in the included literature studies.

## 3. Discussion

Oral drug administration is the preferred route of administration in the pediatric population. Since children, especially younger populations such as neonates and infants, are often in a non-fasted/fed state when these drugs are taken, the effect of food on the disposition of the drug should be evaluated. To date, most food effect studies are performed in the adult population according to health authority guidance, where the composition of the food is not adapted to represent common meal types and quantities for the different pediatric age groups [3]. To truly evaluate these food–drug effects, clinical studies in pediatric populations should be performed. However, it is ethically debatable if the additional information and the associated benefit obtained from these studies can be counterbalanced with the potential burden that these studies pose to the pediatric patients. Therefore, it is worthwhile to explore alternative approaches. In this respect, this current study evaluated whether popPK in combination with food effect studies in beagle dogs and human adults using meal types and quantities representative for the pediatric population could be interesting alternatives to extrapolate food–drug effects to the pediatric population. Moreover, the study focused on the age-appropriate/representative prandial states. 

The popPK of oral administered paracetamol has been described in the literature using a one-, two- or three-compartment model [20,21,22,23]. Therefore, it was decided to start the modelling exercise using a one-compartment model with a first-order absorption. In the beagle dogs, a two-compartment model with subsequent zero- and first-order absorption captured the data better, whereas in the human adults a one-compartment model could be retained. Moreover, when evaluating the plasma concentration–time curves in the human adults, a biphasic absorption profile could be observed in some of the individual profiles (Figure 1). Therefore, a dual input function (Bio and 1-Bio) was included in the model to better capture the shouldering observed in the human adult paracetamol profiles. Furthermore, a lag time for the second input function was modelled, indicating that part of the gastrointestinal absorption of the paracetamol did not start immediately after oral administration. The latter was similar to a study performed by Anderson et al., where a lag time was taken into account to model paracetamol PK in neonates, infants, and children [24,25]. The biphasic absorption profiles might be explained by factors related to formulation (e.g., excipients or the chemical entity itself) and/or the physiology of the gastrointestinal tract (e.g., the pH, anatomy, bile secretion, and discontinuous gastric emptying processes) [26]. Statelova et al. [16] attributed this phenomenon to the variability of the gastrointestinal tract contraction patterns and the viscosity-enhancing excipient (i.e., xanthan gum) present in the paracetamol formulation, leading to insufficient dispersion of the formulation in the stomach and, consequently, non-continuous gastric emptying. This trend could not be found in the plasma concentration–time curves of the beagle dog and the studied pediatric population, which is possibly related to physiological differences in gastric emptying and/or to a lesser extent to the gastric pH [27,28]. The latter was also described by Anderson et al., where the absorption half-life following the oral paracetamol administration was prolonged in the youngest age group (<3 months) in comparison to the older children. The authors attributed this phenomenon to the immature gastric emptying processes, which are slower and more irregular in neonates and young infants [24,25]. 

In both species a significant covariate effect of the WT on the V_d_ and CL could be retained. In the model, the influence of the WT was implemented using a power function with fixed exponents (1 and 0.75 for V_d_ and CL, respectively). Including this covariate effect was physiologically reasonable and was comparable with what was modelled in other popPK paracetamol models found in the literature [20,21,24,25]. Different covariates accounting for food effects (the fed state of the adult human and the beagle dog; the caloric content in the beagle dog) were evaluated during the popPK modeling. No significant food effect could be retained during the covariate analysis in both species, implying that for both the RM and the IF, the food effect at the population level is comparable. Since popPK is data-driven, the lack of a significant food effect could be explained by the design of this study, which might have been underpowered to evaluate these food-related covariate effects on a population level. However, when visually inspecting the plasma concentration–time curves and analyzing the post hoc PK parameters, differences between the different treatments could still be discerned, especially when looking at the data in the beagle dogs. Therefore, it was decided to capture some of these treatment-related differences in the popPK model, by including the IOV on the different absorption-related PK parameters, assuming that the individual parameters vary between the different occasions. Modelling the IOV partly captured the random variability, which was not attributed to the IIV, in the absorption parameters between the different dosing occasions. The latter model was subsequently used for simulation purposes.

The beagle dogs and the human adults were able to adequately simulate the pediatric paracetamol concentration–time curves (Figure 4). The simulation results obtained using popPK were comparable with the results retrieved from a similar PBPK exercise performed by Statelova et al. [14,15]. In this current analysis, for the simulations starting from the human adult results, it was decided to use the popPK parameters, scaled allometrically, since it is known that body weight is an important covariate on PK parameters. This simulation strategy was also applied for the beagle dogs; however, these predictions clearly overpredicted for all the dosing conditions, which was attributed to differences in the CL between the dogs and the humans (Table 3). Therefore, additional simulations were performed, where the absorption-related parameters obtained from the beagle dogs were used but combined with the CL and V_d_ values predicted for the human adults, which were scaled allometrically. The plasma concentration–time curves obtained with these simulations resulted in a significantly better description of the data. When compared to the PBPK exercise, better simulation results were obtained when caloric-based scaling of gastric emptying for the different dosing conditions was implemented for both species. This is an important difference between both tools, where the advantage of PBPK is that one can mechanistically adapt the parameters of the model based on known effects on the gastrointestinal system, i.e., the caloric content, physiological parameters, etc., whereas popPK is data-driven and effects can only be included when they can be directly extracted from the data. 

As shown in this study, PopPK is a powerful tool to perform extrapolations; however, this tool also has its limitations. As mentioned earlier, popPK is data-driven, whereby the data needed to construct and to validate the models are key to perform successful modeling and simulation exercises. For the construction of the model, the data have to be collected considering the specific study objectives, followed by the performance of an exploratory data analysis and data cleaning when required. For the predictive capacity of the developed popPK models, the quality of the data in the external dataset to validate the results is important. In this respect, the pediatric observed data retrieved from the literature were rather scarce (only two studies were retrieved that met the population-/study-specific inclusion criteria) [18,19], did not mention the prandial state, and covered a large age range of the pediatric population. Therefore, an accurate validation of the simulated data was difficult to achieve, so that additional studies mimicking the study design of this present study would still be needed in order to truly validate the results. Furthermore, since both a crossover (in the human adults) and a sequential (in the beagle dogs) study design were applied, it was difficult to disentangle the potential occasion and treatment effects using popPK. To truly evaluate these effects, in future studies it might be opted to use a crossover design for all species, where all individuals receive the different meal types at different occasions but are randomly allocated to the different meal types within one occasion.

The use of the beagle dog had its limitations to reliably predict the pediatric food-effects using popPK for the following reasons. First, anatomical and physiological species differences could have biased the popPK modeling, i.e., differences in motility patterns, intragastric pressure, etc. Second, in the present design it was decided to use adult beagle dogs (with a median age of 1.9 years) to test the predictive performance of the species. However, it is generally accepted that maturation of physiological processes is still ongoing at ages under 2 years, which are more representative for the current case. To evaluate the impact of maturation of the physiological processes on drug disposition, PBPK could be used. If this impact is significant, it might be of interest to use juvenile dogs, more closely mimicking a representative pediatric population, to predict pediatric food–drug effects [29,30,31,32]. Each animal model has its advantages and disadvantages, which should all be carefully considered so that researchers will select the proper model to evaluate the intended study objectives. In addition, in food effect studies performed during drug product development for adult indications, animal models such as the beagle dog are commonly used and have proven to be useful [33,34]. However, the use of juvenile beagle dog models within pediatric drug development and, consequently, the translation to the pediatric population is still rather uncommon [3].

## 4. Materials and Methods

### 4.1. Study Data

#### 4.1.1. Beagle Dog

**Figure 5:**
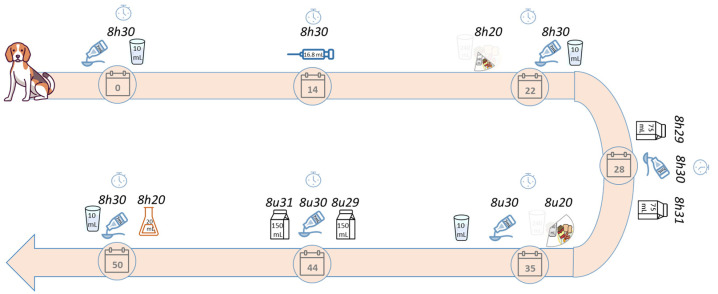
Detailed study design of the paracetamol beagle dog study (adapted from [14]). On days 0, 22, 28, 35, 44, and 50, 7 mL of paracetamol (Panadol^®^, 168 mg paracetamol) was administered to the beagle dogs via oral gavage followed by 10 mL of tap water. The homogenized reference meal was administered on days 22 (RM200) and 35 (RM100). The infant formula was administered one minute before and one minute after the paracetamol administration on days 28 (IF100) and 35 (IF200). On day 50, a 0.1M HCl/KCl solution (pH 1.6) of pretreatment was given ten minutes prior to the paracetamol administration. On day 14, a paracetamol intravenous injection (16.8 mL, 168 mg of paracetamol) was given.

The execution of the beagle dog study was in full accordance with European [35] and national legislations [36]. Ethical committee approval was obtained from the ethical committee of Janssen R&D (approval number 512). A detailed description of the beagle dog study can be found in Statelova et al. [14]. Six clinically healthy beagle dogs (Marshall BioResources, Lyon, France) were included in this present study with a median age of 1.9 years (a minimum of 1.7 and a maximum of 4.0 years) and a median body weight of 9.70 kg (a minimum of 7.90 and a maximum of 13.3 kg). During the acclimatization period, the beagle dogs were group-housed in standard dog cages, where once daily canine dry pellets (LabDiet^®^, St. Louis, MO, USA) and ad libitum drinking water were provided. The beagle dogs were individually housed during the drug administration until 4 h post-dose, after which the beagle dogs were regrouped and could go outside. The study design is detailed in Figure 5. Jugular venipunctures were performed to collect venous blood samples (2 mL) in EDTA-K2 Vacutainers^®^ (Becton, Dickinson U.K. Ltd., Berkshire, UK). The blood samples were kept on ice followed by centrifugation (10 min, 9000× *g*, 5 °C). Aliquots of the samples were stored at −20 °C in amber screwcap microtubes. The paracetamol plasma concentrations were determined using a validated High-Performance Liquid Chromatography Ultraviolet detection (HPLC-UV) method [16]. The lower limit of quantification was 7.5 ng/mL.

#### 4.1.2. Human Adults Study

**Figure 6:**
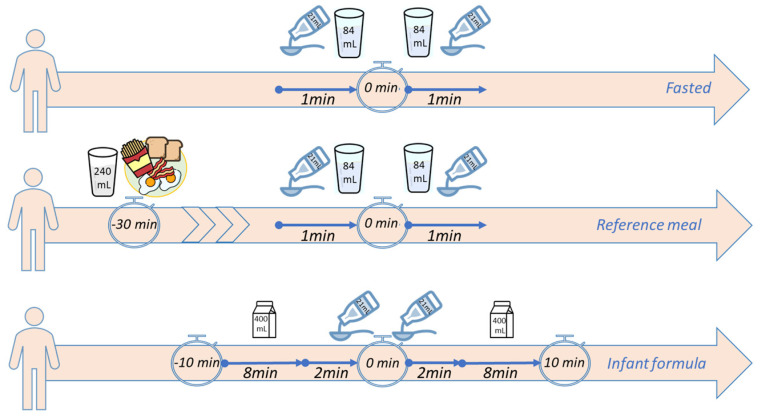
Detailed study design of the paracetamol study in human adults (adapted from [16]). Fasted: 84 mL water and 21 mL paracetamol (Panadol^®^, 168 mg paracetamol) administered twice over 1 min; reference meal: 30 min prior to the paracetamol administration (similar as fasted), the reference meal (240 mL of whole cow’s milk, two eggs, two strips of bacon, two slices of toast, and 56 g of French fries) was administered; infant formula: infant formula (Noulac^®^, 800 mL in total) was administered over 8 min, followed by 21 mL of paracetamol administration over 2 min (twice) and the infant formula again over another 8 min.

Paracetamol was administered to ten healthy adult males in a single-dose, open-label, randomized, crossover, three-period comparative oral bioavailability study. A comprehensive description of the human adults study can be found in Statelova et al. [16]. The detailed study design and the descriptive statistics are depicted in Figure 6 and Table 4, respectively.

### 4.2. Model Development

A non-linear mixed effects model analysis was conducted for the PK parameter estimation using the Monolix software (See Section 4.5). The stochastic approximation expectation maximization (SAEM) algorithm implemented in the software was used throughout the model building. Before the model analysis, data below the limit of quantification (beagle dogs: 1.19%; human adults: 0.83%) were excluded from the dataset. The model building was initiated using a one-compartment PK model with first-order absorption and linear elimination. Subsequently, various absorption models were tested to further improve the description of the absorption phase of the paracetamol. These models included adding parallel/sequential zero-order combined with first-order absorption inputs, lag times, and transit compartments. Discrimination between the models was performed based on a significant drop in the objection function value (OFV, −2 times the log-likelihood), diagnostic goodness-of-fit plots, and the precision of the parameter estimates. A minimal decrease in the OFV of 3.84 points, corresponding to a significance level of *p* < 0.05 (χ^2^ test, df = 1), was considered to hierarchically discriminate between the models. The between-subjects variability (BSV) was estimated for all the PK parameters and assumed to be log-normally distributed. The residual variability was described using an additive-plus-proportional error model. The relevant covariates evaluated in the current study were the body weight (WT), age, food state (fasted vs. fed), and caloric content of the food. Covariate–parameter relationships were first evaluated graphically and then with statistical analysis using Pearson’s correlation test and ANOVA. The body weight was included as a covariate in the model based on allometric principles (fixed allometric exponent):

(1) $$log\left(\theta_{i}\right)=\log\left(\theta_{70pop}\right)+\beta \times \log\left(\frac{WTi}{70}\right)+\eta_{i}+\eta_{occ,i}$$

with θ_i_: individual PK parameter estimate; θ_70pop_: typical population value for the PK parameter in a 70 kg individual; β: allometric exponent; WT_i_: body weight of the ith individual; η_i_: between-subjects random effect; and η_occ,i_: between-occasion random effect. The allometric exponent (β) was fixed to 0.75 for the clearance and intercompartmental flow (CL, Q) and 1 for the volumes (V_d_, V_p_). 

The covariate selection was performed in a stepwise manner, where covariates with a significant impact on the model parameters were retained in the model (forward addition: decrease in the OFV of at least 3.84 points (*p* < 0.05); backward elimination: increase in the OFV of a minimal of 6.635 points (*p* < 0.01)).

### 4.3. Model Evaluation

A visual inspection of the goodness-of-fit plots for the final population PK models was performed to evaluate how well the model describes the data: (1) observed concentrations versus individual (IPRED) and population (PRED) predicted concentrations; (2) individual weighted residuals (IWRES) versus time and versus concentrations; (3) prediction-corrected visual predictive checks (pcVPC), with 500 simulations of the original dataset. These diagnostic plots were stratified by the dosing condition to ensure an adequacy of fit across these conditions. The parameters were considered precisely and estimated when the relative standard error (RSE%) was below 30% and 50% for the fixed and random effects, respectively.

### 4.4. Simulations

The final popPK models were used to understand whether beagle dogs and/or human adults could mimic the food effects observed in the pediatric population [18,19]. During the simulations based on the beagle dog models, the CL and V_d_ values obtained in the human adults and scaled allometrically to pediatrics were used in combination with the absorption-related parameters estimated for the beagle dogs. By contrast, for the simulations in pediatric populations starting from the human adult models, the CL and V_d_ (allometrically scaled) as well as the absorption-related parameters (fixed) of the human adults were used. Monte Carlo simulations were conducted based on the established final models to generate the plasma concentration–time profiles for each dosing condition (*n* = 1000 per dosing condition (dose: 15 mg/kg)) and the age and WT distribution, with a uniform WT distribution for the pediatric population. For the PK parameters where a significant food covariate effect was lacking, the mean post hoc individual PK values per treatment arm were again used accounting for the BSV and IOV. The plasma concentration–time profiles for the paracetamol based on the simulations done using beagle dogs or human adults derived absorption-related parameters, combined with allometrically scaled human adult values for the V_d_ and CL, which were plotted and compared to the data observed in Hopkins et al. [18] (suspension, 15 mg/kg) and Walson et al. [19] (elixir, 12 mg/kg).

### 4.5. Software

Webplotdigitizer was used to digitize the data from the plots in the retrieved literature studies. The dataset preparation, exploratory data analysis, and plot generation were performed using R version 3.6.2 (R core Team; R-project.org, Vienna, Austria). Monolix version 2019R2 (Lixoft SAS, Antony, France) was used for the model development and parameter estimation, whereas NONMEM version 7.6 (Icon Development Solutions, Ellicott City, MD, USA) was used for simulating purposes.

## 5. Conclusions

To conclude, the main objective of this current study was to assess whether beagle dogs and human adults could reliably predict food effects in a pediatric population using popPK. To test this hypothesis, studies where paracetamol was administered to both species under different dosing conditions (fasted, RM, and IF) were conducted. Moreover, different meal compositions (with differing meal quantities and caloric content) were evaluated in the beagle dogs group to mimic a real-life pediatric situation. This current study, using popPK, demonstrated that the studies of both beagle dogs and human adults were able to adequately predict the pediatric paracetamol plasma concentration–time curves. Nevertheless, these results should be carefully interpreted since an accurate validation of these findings was not possible due to the scarcity of the observed pediatric data in the literature. Moreover, these findings should be cautiously extrapolated to age ranges below the current study population.

## Figures and Tables

**Table 1:** Pharmacokinetic parameters (mean ± standard deviation) for administration of the paracetamol to six beagle dogs under different dosing conditions.

	AUC_0-inf_ (µg·h/mL)	C_max_ (µg/mL)	T_max_ (h) ^a^
Fasted	9.86 ± 1.19 ^1^	6.07 ± 1.46 ^5,6^	0.63 [0.25–1.00] ^13,14,15^
Fasted HCl/KCl	11.4 ± 1.06 ^2^	6.11 ± 1.55 ^7,8^	1.00 [0.50–1.50] ^16,17,18^
RM100	11.6 ± 1.23 ^1,3^	6.21 ± 1.42 ^9,11^	0.88 [0.75–1.00] ^19,21,22^
RM200	9.86 ± 1.16 ^2,3,4^	4.50 ± 0.99 ^5,7,9,10^	1.50 [1.00–1.50] ^13,16,19,20^
IF100	16.2 ± 7.15	5.18 ± 0.86 ^12^	1.50 [1.00–2.00] ^14,17,21^
IF200	11.3 ± 1.49 ^4^	3.79 ± 0.76 ^6,8,10,11,12^	1.75 [1.50–2.50] ^15,18,20,22^

AUC_0-inf_: Area Under the Curve extrapolated to infinity; C_max_: maximum plasma concentration; T_max_: time to maximum plasma concentration; RM: reference meal; IF: infant formula; RM200: 100 g (200 kcal); RM100: 50 g (100 kcal); IF100: 150 mL (100 kcal); and INF200: 300 mL (200 kcal). Results with the same numerical superscript are considered significantly different (repeated measure ANOVA, *p*  <  0.05). ^a^: median [range].

**Table 2:** Pharmacokinetic parameters (mean ± standard deviation) for administration of the paracetamol to adults (*n* = 8) under different dosing conditions (adapted from [16]).

	AUC_0-inf_ (µg·h/mL)	C_max_ (µg/mL)	T_max_ (h) ^a^
Fasted	39.3 ± 9.53	7.85 ± 1.54	1.50 [0.33–4.00] ^1^
Reference meal	40.2 ± 10.7	6.96 ± 2.42	2.50 [0.75–5.00]
Infant formula	39.2 ± 10.1	7.24 ± 1.32	4.00 [1.50–5.00] ^1^

AUC_0-inf_: Area Under the Curve extrapolated to infinity; C_max_: maximum plasma concentration; T_max_: time to maximum plasma concentration; reference meal: 990 kcal; and infant formula: 520 kcal. Results with the same numerical superscript are considered significantly different (repeated measure ANOVA, *p*  <  0.05). ^a^: median [range].

**Table 3:** Final model parameter estimates after administration of paracetamol to the beagle dogs and the human adults.

Parameter	Estimate Beagle Dogs [RSE%]	Estimate Human Adults [RSE%]
Structural model		
F	0.80 [2.56]	-
Bio	-	0.45 [27.5]
k_a_ (h^−1^)	2.86 [14.2]	1.79 [38.1]
V_d_ (L)	9.53 [1.55]	27.6 [2.42] *
CL (L/h)	9.29 [1.92]	8.79 [1.99] *
V_p_ (L)	34.9 [4.56]	-
Q (L/h)	2.82 [0.835]	-
dT1 (h)	0.64 [16.1]	0.22 [39.2]
dT2 (h)	-	2.73 [34.2]
T2 (h)	-	0.97 [31.8]
Covariate model		
β_WTonCL_	0.75 [FIX]	0.75 [FIX] *
β_WTonVd_	1 [FIX]	1 [FIX] *
β_WTonVp_	1 [FIX]	-
β_WTonQ_	1 [FIX]	-
Between-subjects variability		
BSV V_d_	-	0.098 [39.9] *
BSV CL	-	0.13 [26.0] *
Inter occasion variability		
IOV F	0.11 [14.3]	-
IOV Bio	-	0.69 [20.7]
IOV k_a_	0.63 [18.0]	0.53 [56.4]
IOV dT1	0.86 [15.2]	1.58 [25.9]
IOV dT2	-	0.87 [22.0]
IOV T2	-	0.59 [38.3]
Error model parameters *^1^		
a	0.055 [9.30]	0.094 [23.9]
b	0.13 [7.13]	0.094 [11.2]
Log-likelihood estimation		
−2LL	135.01	659.71
AIC	159.01	691.71
BIC	179.86	710.56

* V_d_/F, CL/F; *^1^ combined proportional and additive error model; F = oral bioavailability; bio = fraction of the absorbed dose; k_a_ = first-order absorption rate constant; V_d_ = volume of distribution; CL = total body clearance; V_p_ = volume of distribution of the peripheral compartment; Q = intercompartmental flow; dT1 = duration of zero-order input into dosing compartment 1; dT2 = duration of zero-order input into dosing compartment 2; T2 = fractional lag time; T_lag_ = lag time; β = covariate effect coefficient; WT = body weight; BSV = between-subjects variability; IOV = inter occasion variability; a = additive error model term; b = proportional error model term; −2LL, −2 times log-likelihood; AIC: Akaike Information Criterion; BIC: Bayesian Information Criterion; and RSE = relative standard error.

**Table 4:** Descriptive statistics of the study in human adults (adapted from [16]).

Variable	Value
No. of healthy volunteers	8 *
Age, years (median [min–max])	25 [21–48]
BMI, kg/m^2^ (median [min–max])	23.8 [20.3–27.7]
Height, m (median [min–max])	1.85 [1.69–1.92]
Body weight, kg (median [min–max])	81.5 [60–104]
Sex	Male
Race	Caucasian
Dose, mg	1000 (42 mL Panadol^®^ suspension, 24 mg/mL paracetamol)
PK samples	15
PK sampling times	0–10 h

* Ten healthy adult volunteers were included, but only eight completed the study. BMI: body mass index; PK: pharmacokinetics.

## Data Availability

The data presented in this study are available on request from the corresponding author.

## Supplementary Materials

The following supporting information can be downloaded at: https://www.mdpi.com/article/10.3390/ph17010053/s1. Figure S1: individual fits for paracetamol in six beagle dogs (upper panel; the blue dots represent the observed data with the lines representing the different occasions. Blue: intravenous/fasted; red: per os/fasted no HCl pre-treatment; yellow: per os/reference meal 200 (100 g, 200 kcal); green: per os/infant formula 100 (150 mL, 100 kcal); purple: per os/reference meal 100 (50 g, 100 kcal); light blue: per os/infant formula 200 (300 mL, 200 kcal); and pink: per os/fasted HCl pre-treatment) and eight human adults (lower panel; the blue dots represent the observed data with the lines representing the different occasions. Blue: per os/fasted; red: per os/reference meal (990 kcal); and yellow: per os/infant formula (2 times 400 mL (260 kcal)). Figure S2: scatterplot of the observed plasma concentrations versus the population (left) and the individual (right) predicted plasma concentrations of paracetamol in beagle dogs (a) and human adults (b). Blue circles represent the observed data. The full black line is the line of unity; the full red line is the LOESS smoothed line; and the dotted lines are the 90% prediction intervals. Figure S3: scatterplots of the individual weighted residuals (IWRES) versus the time since the last dose and population predictions (Cc) in beagle dogs (a) and human adults (b). Blue circles represent the observed data. The dashed line is the zero line, and the full red line is the LOESS smoothed line.
