# Supplementary material for: The Use of Population Pharmacokinetics to Extrapolate Food Effects from Human Adults and Beagle Dogs to the Pediatric Population Illustrated with Paracetamol as a Test Case
Source: Pharmaceuticals (Basel). 2023 Dec 28;17(1):53. doi: 10.3390/ph17010053 (PMC10818831; doi:10.3390/ph17010053)
Supplement: Supplementary file 1 [file pharmaceuticals-17-00053-s001.zip › pharmaceuticals-2775788-supplementary.pdf]

Supplementary Materials

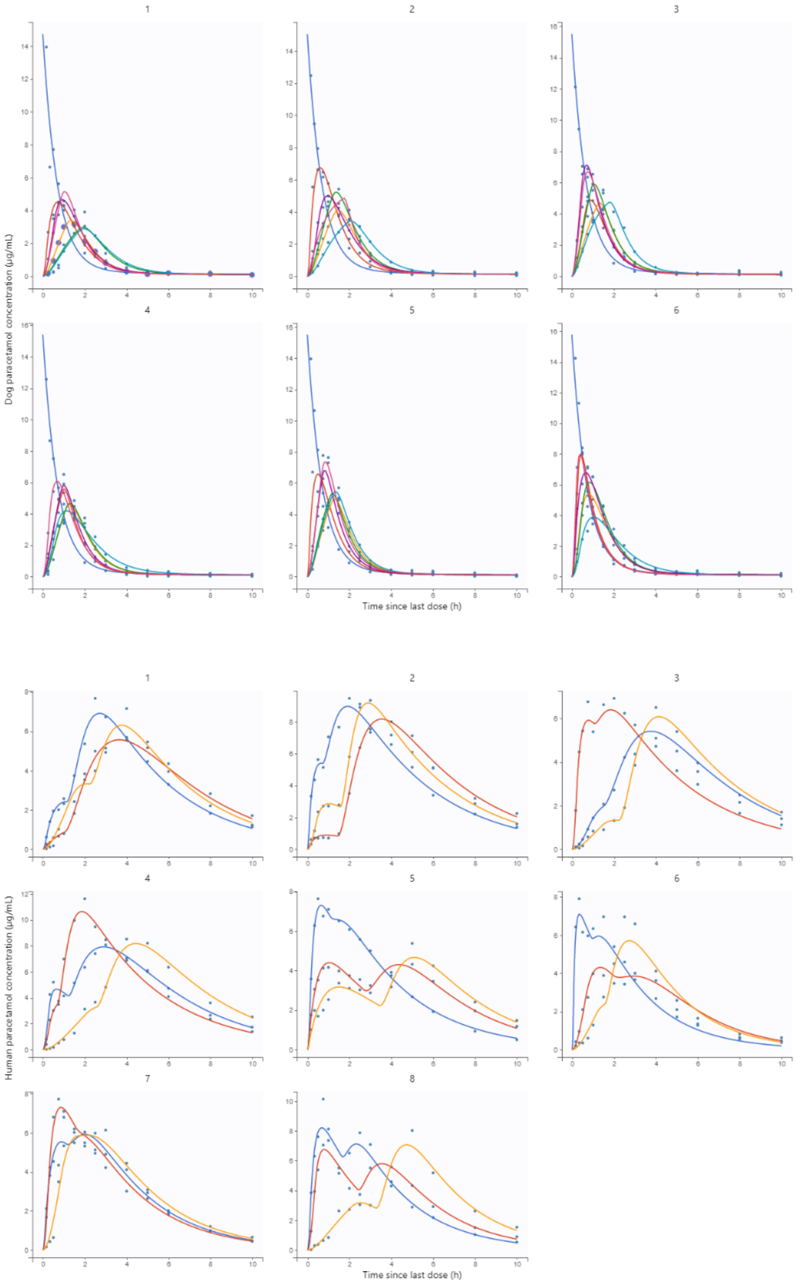

Figure S1. Individual fits for paracetamol in 6 beagle dogs (upper panel, blue dots: observed data, lines represent the different occasions: blue: intravenous/fasted; red: per os/fasted no HCl pre-treatment; yellow: per os/reference meal 200 (100 g, 200 kcal); green: per os/infant formula 100 (150 mL, 100 kcal); purple: per os/reference meal 100 (50 g, 100 kcal); light blue: per os/infant formula 200 (300 mL, 200 kcal); pink: per os/fasted HCl pre-treatment) and 8 human adults (lower panel, blue dots: observed data, lines represent the different occasions: blue: per os/fasted; red: per os/reference meal (990 kcal); yellow: per os/infant formula (2 times 400 mL (260 kcal)).

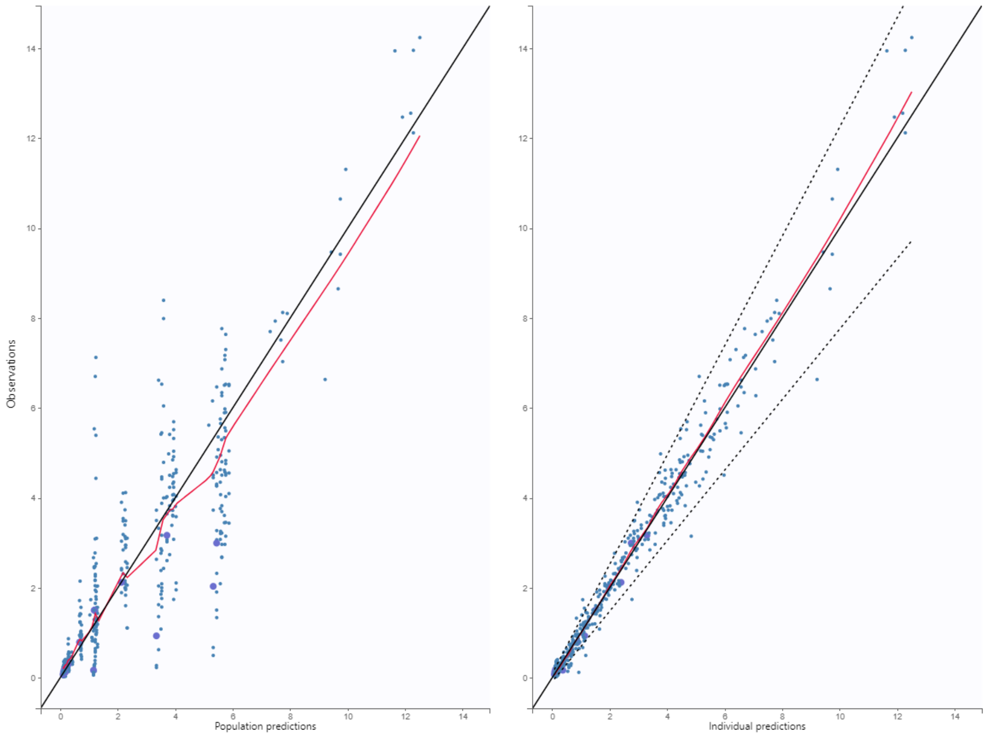

(a)

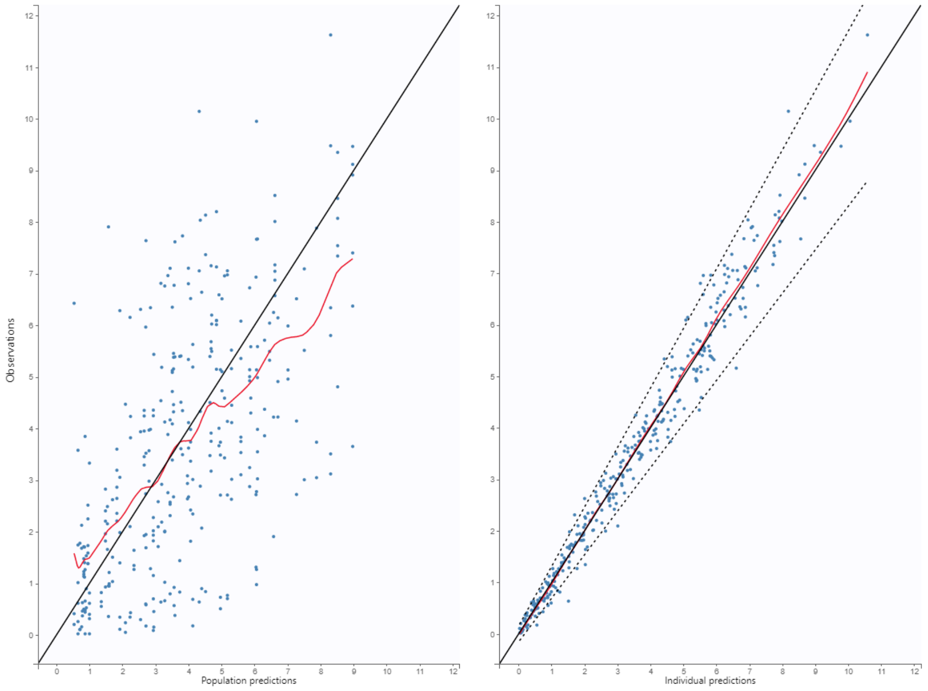

(b)

Figure S2. Scatter plot of the observed plasma concentrations versus the population (left) and individual (right) predicted plasma concentrations of paracetamol in beagle dogs (a) and human adults (b). Blue circles represent the observed data, the full black line is the line of unity, the full red line is the LOESS smoothed line, the dotted lines are the 90% prediction intervals.

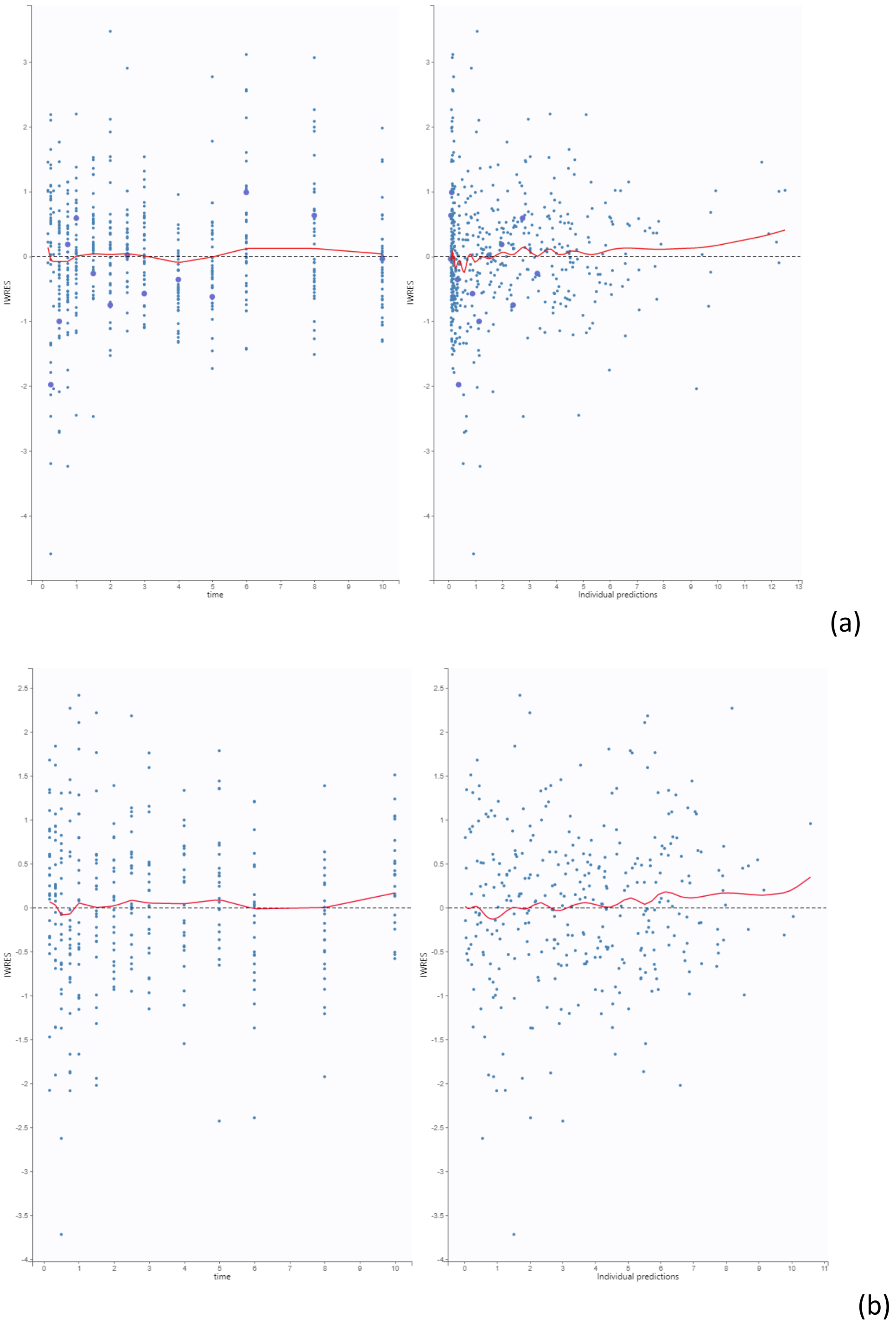

Figure S3. Scatter plots of the individual weighted residuals (IWRES) versus time since last dose and population predictions (Cc) in beagle dogs (a) and human adults (b). Blue circles represent the observed data, the dashed line is the zero line, the full red line is the LOESS smoothed line.
